# *Pasung*: A qualitative study of shackling family members with mental illness in Indonesia

**DOI:** 10.1177/13634615221135254

**Published:** 2022-11-24

**Authors:** Børge Baklien, Marthoenis Marthoenis, Arif Rahman Aceh, Miranda Thurston

**Affiliations:** 1 3207 Inland Norway University of Applied Sciences (INN), Norway; 2 Department of Psychiatry and Mental Health Nursing, 175503 Universitas Syiah Kuala, Indonesia; 3 188395 STIKes Flora, Indonesia

**Keywords:** coercion, depersonalization, Indonesia, mental illness, pasung

## Abstract

Use of coercion on people with mental illness is a deeply embedded practice around the world. Not only does the practice raise human rights issues, it also leads to further mental, physical, and emotional harms. In Indonesia, ‘pasung’ is a common practice of physical restraint, which involves lay people using a variety of illegal methods to tie a person. In this article, we explore the meanings families attach to their actions when using pasung by asking the question: to what extent does the use of pasung by families emerge from socioculturally prescribed norms and conventions? To explore this question, we conducted and analysed eight interviews with family members from Nias Island, Indonesia using Giorgi's descriptive phenomenological method. Our findings reveal that pasung emerges in the disjunction between sociocultural demands and the family's capacity to meet these demands. Struggling to understand the behaviour of a family member with mental illness, the family tries to cope with neighbourhood reactions to ever more visible behavioural signs alongside managing their everyday life. These struggles, in turn, make their social situation increasingly stressful, which initiates a process of depersonalization as a response. Moreover, the prevailing sociocultural values convey a need to act according to expected norms. As such, pasung materializes as a socioculturally accepted practice that allows families to take back control in stressful social situations. In sum, when families feel overwhelming emotional stress and a sense of powerlessness, they try to resolve their situation by using pasung to regain control and thus manage their lives.

## Introduction

Caring for relatives with a serious mental illness in everyday life presents an enormous challenge for families around the world. For too many families, the use of physical restraint and confinement seems the only possible strategy. Hundreds of thousands of women, men, and children with mental illness across many countries have, at least once in their life, been shackled in unhygienic conditions with limited access to a toilet and food (Sharma & Human Rights Watch, 2020). Accordingly, many vulnerable people have been locked up for several years, never being let outside (Sharma & Human Rights Watch, 2020). In Indonesia, this practice is called pasung, which literally means to tie or bind, and involves a variety of illegal methods used by lay people to tie or chain a person to wooden blocks, using ropes or shackles, or to lock them in rooms, cells, cages, or animal sheds (Molodynski et al., 2016; Puteh et al., 2011). The living conditions for those in pasung can be appalling (Minas & Diatri, 2008).

The practice continues even though The Convention on Rights of Persons with Disabilities (CRPD) was ratified by Indonesia in 2011. It sought to guarantee equal rights, security, and freedom from torture and mistreatment, yet coercion in the form of pasung remains a significant problem. This deeply embedded social practice not only undermines people's human rights but also leads to further mental, physical, and emotional harms. Notably, the prevalence of mental illness together with few treatment and support options has meant that the use of pasung by families has become widespread, especially in rural areas and among families of low socioeconomic status (Irwanto et al., 2020). Although accurate data on its prevalence in Indonesia is difficult to obtain, a relatively recent (2018) estimate indicated that 17% of rural households (10.7% in urban areas) with a member living with a psychotic disorder had used pasung with that individual at some point during their lifetime (Prastyani, 2019). A recent estimate suggests that there may be as many as 81,300 cases of pasung in Indonesia (Ilmy et al., 2020a). Even though statistics likely under-estimate its use due to the stigma and shame attached to having a relative with mental illness, they nonetheless are indicative of it being commonplace.

In spite of its well-documented harms, family members initiate the use of pasung and, in some instances, its use is supported or even imposed by community leaders (Anto & Colucci, 2015; Broch, 2001; Minas & Diatri, 2008; Puteh et al., 2011; Suryani et al., 2011). Little is known, however, about the dynamics of how families come to use pasung or their intentions in so doing, which is the departure point for this article. Because pasung has become an embedded sociocultural practice in Indonesia, families may have little awareness or comprehension of the harms associated with its use nor view their actions as in any way damaging. However, beyond the sociocultural milieu of families and communities, such actions can seem intentionally harmful and perhaps even be used as punishment.

In this article, we explore the meanings families attach to their actions when using pasung by asking the question: to what extent does the use of pasung by families emerge from socioculturally prescribed norms and conventions? To explore this question, we examine family members’ first-person experiences of using pasung with a relative, in order to try to understand and make sense of their experiences. Accordingly, our inquiry is phenomenological in that we approach their experienced reality as authentic (Hamblet, 2014, p. 21). Using pasung is thus seen as part of the context in which actions are bound up with meaning (Csordas, 2019, p. 42). Furthermore, the practice is a phenomenon that is subjected to various interpretations because “events are not just there and happen, but they have a meaning and happen because of that meaning” (Weber in Geertz, 1973, p. 131). With this understanding of pasung we approach the phenomenon with empathic understanding of families’ actions—seeking to bracket out our culturally embedded values and norms relating to the harms of pasung—to describe how the act is meaningful for them. In other words, we accept that the phenomenon is experienced and perceived differently for those we are studying. In sum, our argument is that rather than blaming families, there is a need to start with an understanding of their sociocultural situation and the possible coping strategies they fall back on for managing relatives with mental illness. We start by describing the sociocultural context in Indonesia as it relates to mental illness and the use of pasung.

## Pasung, stigma, and mental health in Indonesia

Indonesia has a population of over 270 million people and comprises 17,000 islands. It is a middle-income country where access to good-quality mental health care—including ongoing medication—is relatively weak, and this is especially the case in rural areas, which suffer from an unequal distribution of health workers that favours urban areas (Prastyani, 2019). While Indonesia has 48 psychiatric hospitals and 269 psychiatric wards in general hospitals, demand exceeds capacity, and some provinces are without any psychiatric service or support (Pols, 2020).

Moreover, hospitals tend to be overcrowded and have poor conditions, which themselves can give rise to disrespect for patients’ human rights through the use of control measures. In the same vein, hospitals have insufficient numbers of appropriately trained psychiatric and mental health care staff. Although it is difficult to obtain accurate and up-to-date statistics on the mental health care system, there is some indication that the primary care system is improving. Hunt et al. (2021), for example, describe the strengthening of mental health care teams, the training of general practitioners, as well as increases in the numbers of mental health nurses and psychiatrists. Given that these services are covered by the national insurance scheme, they go some way to extending access to better-quality mental health care services. However, given the prevalence of psychosocial disabilities in Indonesia and the prevalence of pasung, there is still some way to go (Hunt et al., 2021; Pols, 2020), especially with regard to psychiatric hospitals, several of which are overcrowded, with unsanitary conditions, physical violence against patients, and which use forced seclusion to “discipline” the patients (Sharma, 2016). Some indication of the mismatch between capacity and demand is indicated by the numbers of health professionals: approximately 1,000 psychiatrists and 7,000 community mental health nurses for a population of 270 million (Pols, 2020).

The improvements identified above reflect the recent policy developments in Indonesia, which indicate that there is some political momentum for building a better mental health care system (Prastyani, 2019). The 1966 mental health law aimed to protect the rights of the mentally ill, including their right to rehabilitation and treatment, thus making pasung technically illegal. However, it was not until 1977 that using pasung was specifically banned on the basis that it was an “inhumane” and “discriminatory” treatment of people, which violated their fundamental human rights (Wu et al., 2016). Since then, the government has launched several programs to promote mental health and end pasung, including a program called *Indonesia Free from Pasung 2014* (Irmansyah, 2019b), which has now been extended to 2030. Consequently, several regions have legislated to end pasung practices and a number of provinces have specific programmes aimed at finding, freeing, and treating people who have experienced pasung (Hunt et al., 2021; Irmansyah, 2019a, 2019b).

To date, there has been limited research on pasung, but what seems to be emerging is evidence of the considerable burden it places on families and the communities in which they live (Hidayat et al., 2020). Many Indonesian families are poor (Laila et al., 2018, 2019), yet they are compelled to take responsibility for relatives with mental illness according to sociocultural “rules” (Buanasari et al., 2018) and established norms that extend from the micro to the macro level of society. Moreover, family members have limited knowledge and understanding of mental illness, which can also be the case among community leaders (Laila et al., 2018; Subu et al., 2021). In this context, they try to manage the double burden of coping with often aggressive and destructive behaviours while trying to ensure the safety of the whole family and wider community (Ilmy et al., 2020b).

Family members often experience pressure from people in the community to act and control relatives with mental illness, who are often perceived as dangerous (Hall et al., 2019; Hartini et al., 2018). Consequently, if mental illness is interpreted culturally as “craziness” (Broch, 2001; Irwanto et al., 2020) or as being possessed or harmed by spirits, or being exposed to magical practices or forces intended to harm a person (Good & Subandi, 2004; Patawari et al., 2020), then “treatment” from diverse traditional or religious healers might be sought (Yunita et al., 2020). Often these kinds of healers are used alongside medical services (Puteh et al., 2011). In some cases, the family uses all their income on such treatment after pressure from the community and if they find that it does not help, they can feel ashamed and, in some instances, they react by chaining the person (Sharma & Human Right Watch, 2020, p. 36).

The use of pasung therefore emerges from a sense of powerlessness, alongside a lack of money for treatment and poor knowledge of mental health; thus, its use materializes as a necessary response to protect the person with mental illness as well as others from potentially aggressive and harmful behaviour (Laila et al., 2018, 2019). Still, some families find the money to send their relatives to the medical system believing that a cure can be found. However, on returning home the risk of relapse creates a further challenge for families, who then resort to their previous strategies as a way of coping with the difficulties they are confronted with. Given the pervasiveness of beliefs about the curative powers of medicine, this can give rise to feelings of hopelessness (Hunt et al., 2021). Alternatively, the families might choose treatment with non-medical or alternative therapies and neglect prescribed medication (Subu et al., 2021).

In addition, relations between family members and the person suffering with mental illness can deteriorate as negative feelings towards them can develop (Yunita et al., 2020). In this way, Indonesian families—in these kinds of situations—worry about “disgrace” and “shame” (*aib*) related to stigma; yet it is forbidden to disclose the family's *aib* (Prastyani, 2019). Stigma—that is to say, the “undesired differentness” that can result in a “spoiled identity” produced in the interactive space between individuals in a culturally defined social world (Goffman, 1963)—is commonplace in Indonesia (Hartini et al., 2018). It entails families “feeling” pressure to act from community social norms because they are worried about shame (Hartini et al., 2018; Subu et al., 2021). Moreover, stigma can also have an impact on marriage prospects, honour, employment opportunities, and status in the local community (Rugkåsa, 2016).

In the same vein, research indicates that stigma emerges from particular forms of understanding in the community, which tend to endorse beliefs about people with mental illness—for example, as dangerous, and that they should remain inside to avoid causing trouble (Hall et al., 2019; Ilmy et al., 2020b). Not only are they viewed as dangerous, but they are also often seen as lacking capacity, and bullied or subjected to other forms of violence in the process (Hall et al., 2019). These ways of understanding give rise to perceptions that the only way to control aggressive behaviour is through the use of pasung (Irwanto et al., 2020) given the absence of other forms of support. Therefore, the stigma of mental illness can affect the entire household and the extended family and may well lead to a disruption of family and community life if not dealt with (Ulya, 2019).

What can one infer from this literature? It underscores a perceived discrepancy between the social, cultural, physical, and psychological demands of having a family member with mental illness and their resources to cope with such a situation. Therefore, to answer the research question, we have listened to the families themselves and their first-person accounts of their use of pasung.

## Method

### Recruitment of participants

The family members who were using / had used pasung were recruited through the mental health nurses working in the community health centres across the city of Gunungsitoli, which is located on the Nias Island and part of North Sumatra Province of Indonesia. The city had a population of approximately 136,000 in 2020 and is considered the oldest and largest city in Nias Island.

### Data collection

Eight interviews were conducted by the third author, who was originally from the area from which participants were recruited. They were carried out in the local Nias dialect in the families’ homes. Notably, in phenomenological research this number of interviews is generally viewed as generating sufficient data to reveal the meaning structure (Englander, 2012). According to Giorgi (2009), what matters is how many times the phenomenon (using pasung) appears in the descriptions. The interviews started with an open question asking participants to narrate their experiences of caring for a person under pasung, with open follow-up questions to add more richness and detail to the interview. The interviews were audio-recorded and subsequently transcribed verbatim. The second author translated the interviews into English, which was the language in which the analysis was carried out. The project received ethical approval from the Universitas Syiah Kuala Faculty Ethics Review Board research reference code 112017170320. The researcher obtained written informed consent from each participant prior to the interview.

### Phenomenological design and analysis

The study had a phenomenological design as described by Giorgi (2009), which informs the research process from research interest through to the presentation of findings. The purpose of a descriptive phenomenological method is to uncover the meaning structure of an experienced phenomenon as it appears in the consciousness of those who experience it and express it as a consistent statement. In other words, the meaning structure is what makes the phenomenon that very phenomenon it is perceived to be—it describes the lived experience of the phenomenon (Giorgi, 2009). As such, the meaning structure is based solely upon what is in the data and does not include interpretations that are theoretically informed; it is descriptive rather than explanatory. Therefore, the researcher tries to minimize her or his theoretical assumptions about the phenomenon (Giorgi, 2009, p. 127). Consequently, the researcher must suspend or “bracket off” theoretical assumptions during the analysis of the phenomenon and adopt an attitude of wonder about concrete meaning. However, once the analysis has been carried out, the meaning structure of the phenomenon becomes open to further interpretation.

Drawing on Giorgi's (2009) descriptive phenomenological method, we used a five-step process to data analysis. In the first step, each of the eight transcripts was read to develop a sense of the whole concrete description of the experience. In the second step, we sought to adopt the attitude of phenomenological reduction while being sensitive to the specific phenomenon (using pasung) being studied. This means using bracketing to lessen the influence of preconceptions about the phenomenon in order to encounter it freshly. In essence, it involved setting aside the taken-for-granted attitude about pasung and our knowledge about the negative consequences of treating others in ways which undermine human rights. Bracketing also involves setting aside theoretical assumptions as well as the ontological disbelief in magic and accepting that it may exist for those living in the community we studied. Using this kind of thinking, all the transcripts were read with a commitment to the phenomenological attitude to develop a preliminary sense of the whole and to develop some idea of how the description proceeds and ends (Giorgi, 2018, p. 98).

In the third step, the first author re-read the interview transcripts maintaining a phenomenological attitude in order to break the text into meaning units. That is to say, the researcher marked in the text every time a significant shift in meaning was identified. As such, each individual interview transcript was broken down into a series of meaning units consisting of phrases, sentences, or whole passages that made relatively coherent sense. Every meaning unit was edited from first-person statements to third-person statements by replacing “I am very sad” with “P [participant] was very sad.” According to Giorgi (2009), by using third-person statements one avoids mixing the participant's experience and that of the researchers.

The fourth step is a process called imaginative variation, in which the researcher tries out different formulations of each meaning unit at various levels of generalization to articulate an expression that is suitable. This means that we sought to express the implicit meaning in a more direct and explicit form by extracting and reflecting upon each of the delineated meaning units without using the terminology of mainstream psychology. In this way, no coding was undertaken; rather, all the meaning units were interrogated for their relevance and transformed to highlight the psychological meaning attached to using pasung with family relatives by those interviewed. This transformation process is at the heart of the method of phenomenological descriptive analysis where the psychological meaning must be “detected, drawn out and elaborated” (Giorgi, 2009, p. 131). Table 1 illustrates the process.

**Table 1.** Transforming the meaning unit.

Meaning unit	Transformed meaning unit
He doesn't have any insight, but if P lets him go, he can run everywhere like a chicken that has just been released and doesn't know where to go. P wants to free him, but he goes everywhere, and he does not know how to get home. He might go to take a sea bath for himself. P is afraid that anything can happen to him without P's knowledge. ** **	P states that her son does not understand that he is ill and that he cannot take care of himself. Therefore, according to P, he must be locked inside to protect him from himself and from getting lost or from doing risky things. P compares her son's capability to take care of himself with a chicken. P states that even if she wants to release him, she cannot because he will just go everywhere and will not know how to find his way home. P states that without control over her son she feels powerless and that she will constantly be worried about what he will do and what might happen to him.

In the fifth step, the transformed meaning units relating to all eight transcripts were synthesized into the general meaning structure. According to Giorgi (2009, p. 200), it is not “a matter of simply listing the meaning units together but to bring a holistic perspective on them.” In this way, all the participants’ experiences of the phenomenon were included in the general meaning structure. This was done by reviewing all the transformed meaning units and comparing and contrasting them to decide which ones were necessary for describing the general meaning structure of the phenomenon “using pasung.” In other words, this step is a reflective process in which we tried to determine what was essential to the description.

### Findings

The findings relate to the eight participants recruited to the study. The sample was diverse in terms of age and sex: the age ranged from 24 to 73 years old, and half the sample were women. Seven participants were married and one single. In terms of religious affiliation, five were Muslim and three Christian. Five participants were not in employment; two worked as labourers and one as a salesperson. All had attended school, five to the level of high school.

First, we present the final general meaning structure that was built up from all eight transcripts. Then we unpack the three constituents that were interdependently related, which together constitute the general meaning structure. The three constituents elaborate the meaning structure, the latter being the main point of reference, and are explored separately in the sub-sections that follow.

### The general meaning structure of the phenomenon “using pasung”

For the family member (P) the use of pasung occurred when a relative with mental illness (Q) started to behave in a manner that P did not recognize as meaningful and did not understand, and P became unsure what to think or do. P did not recognize Q as the person s/he was before the strange behaviour appeared; it was as if the person P once knew was no longer wholly present. When Q became angry, aggressive, and noisy P became increasingly anxious about the possibility of damage to objects and injuries to people as well as how the neighbours might react. Since P needed to work to economically support the family it was difficult to ensure that Q did not harm her/himself or others—or create any disturbance in the neighbourhood. P started to feel the social situation was stressful and when Q became aggressive or made noise, P's feelings were aroused. P felt a need to act, and P realized that using pasung was a way to stop Q's behaviour. P felt sorrow for Q but had no regret about using pasung since it had allowed P to stop worrying about Q's behaviour in everyday life.

This meaning structure of the phenomenon “using pasung” comprised three constituent parts. The first was that Q was drifting into a differently experienced world with behaviours P did not comprehend or recognize; the second was the increasingly stressful social situation of coping with the behaviour; and the third was not recognizing Q as a person but as behaviours that had to be stopped. These three constituents unfolded over time; they illustrate the dynamic process of the socially situated phenomenon “using pasung.”

#### Constituent 1

##### Drifting into a different world with behaviours the family does not understand

This constituent describes an uncertainty in how the family ought to respond to behaviours that have no meaning for them. Moreover, when their family member started to talk about imaginary things or acted aggressively and loudly, they realized that something was wrong, however they were uncertain what to think or do. Even though the family knew that it was an illness, they had no knowledge that could guide them in how to cope with such behaviours.

When he wanted to leave, he told me why the stars were chasing me, but these words we did not welcome because we knew it was impossible. After that he seems to be hostile to the family at home and he asks us why we are giving him poison. … Those were the first signs of his behaviour. A few months later he destroyed things at home such as cauldrons, pots, light bulbs, mirrors.

The families did not recognize the family member as the person they knew before the illness—it was the illness that had taken command over the person.

Indeed, at that time he apologized and begged not to be tied up, but we said it was not your fault, you did not do it on purpose.

Furthermore, the family described this as losing the family member they knew; they prayed and hoped that the person would return.

I am very sad because my child is the only one I have and love. She is like a crazy person now.

Because of uncertainty over how to cope with undesirable behaviours, the family sought advice from neighbours and local chiefs or help from traditional healers. Even when they acted on advice from the local community or from medical professionals, the behaviour continued.

We started healing at that time using traditional medicine, however it never stopped. Every time he relapses. We wanted to treat him with traditional medicine and maybe there are 28 people [28 different traditional and religious healers] who have treated him in various ways.

But without much help from their local health system their uncertainty—of how to cope with the disturbing behaviour—grew; they decided to take control over the stressful situation by using pasung.

She has been locked up since she disturbed people. Her brother said, just lock her up so that the neighbours don't make a fuss; it has been about six months since her brother and I locked her in the room. The important thing is that she is not behaving strangely.

#### Constituent 2

##### Increasingly stressful social situation of coping with the behaviours

This constituent describes how the family experienced frustration and stress when the family member drifted into a different world and began acting aggressively and loudly. At the same time, they found it difficult to continue their everyday life because their family member needed constant care and attention. In addition, the local community expected them to act in order to prevent their relative from disturbing the community.

Because everyone in this home is working and we cannot look after her. We only look after her upon return from work. … I can't let her go because no one is looking after her, I’m busy working, so if anything happens, I can be reprimanded and have to take responsibility. So, all the problems multiply.

This, in turn, created stress because they struggled to cope with their social situation. As such they experienced a struggle between the difficulties of coping with aggressive and strange behaviour and, at the same time, having to work to provide income for the family. Therefore, they felt uncomfortable leaving the person alone at home and they experienced the situation as particularly stressful when they were unsure what could happen while they were away at work.

I do not want to let her go because there is no one to look after her, especially when I must go to work from morning to evening. Most lunch times I will come back, but I am afraid that something will happen to her.

One of the family's main concerns was living with an economic burden. When the family member with mental illness was not able to work, they started to feel the burden of having to take responsibility for the person. If the person was not getting any better, they continued trying to cope. However, at some point they lost hope, or it just became too expensive to manage things the way they had been doing. Accordingly, the economic burden—alongside their lack of knowledge about mental illness and the limited medical support system—meant that the use of pasung seemed like a strategy that gave the family some respite when they were exhausted.

How many million [rupiah] have I spent … I can no longer add up how much the total cost has been … how much gold I sold just to find traditional medicine for him and some dukuns. I was also tricked [by a traditional healer] who I paid a large amount of money to. Finally, I gave up and now I am not looking anymore for how to treat him.

The families became especially anxious about the neighbours’ reactions and this further aroused feelings of anxiety in an already stressful situation. On the one hand, the families said that they had good relationships with their neighbours and that they understood their situation. On the other hand, however, the families were anxious about their neighbours’ reactions. Therefore, they felt ashamed of having a relative with mental illness; they wanted to avoid any noise or trouble in the neighbourhood.

Once my child cried, she screamed at the cemetery near here, until the agitation of the neighbours called me home from work. Then she was tied up in her room.

The family felt responsible for disturbing other people and were concerned that they should pay for any damage. In addition, they knew that it was expected in the neighbourhood that they should take care of their relatives and prevent them from walking around in the neighbourhood creating a disturbance. As such, they were also nervous about gossip and felt ashamed.

The whole family agreed to put him in that room, because he likes to go everywhere and make a lot of noise, which disturbs people. He went to the shop where he destroyed the goods and there was a scene. Also, the head of the village warned us to look after our child and to look for ways to prevent the child from running around and being noisy.

#### Constituent 3

##### Coming to see the family member not as a person but as behaviours that must be stopped

This constituent describes how the families come to see the person as a behaviour or collection of behaviours that must be controlled. When the behaviour appeared idiosyncratic and irrational to the family—who were unfamiliar with mental illness—the stress of their situation increased; they acted according to the prescribed norms of society.

It is worse if we do not lock her up, right? We want to avoid her doing something bad or disturbing people, especially to many of our neighbours who get angry with her [because of her behaviour].

The family used pasung because they came to see it as a solution that could release them from a stressful situation—by preventing the strange behaviour.

He came home angry and asked for money and destroyed all the things just because he wanted money. He was like a stressed person. Because I had a headache [stressed], I confined him in his room. I tied him up so he wouldn't fight. This is because I am also stressed because my wife is afraid.

Consequently, by using pasung they could take back some control. Moreover, the family had an ongoing feeling of anxiety about the situation even when trying to control things. The family dynamics shifted to trying to contain the problem and the consequences of it for their everyday life. Thus, they had to “live with” using pasung.

No, because this is to keep him from hitting other people, not to torture him. Because if he is angry, whatever is there, whether iron or wood, he hits someone with it, so who handles it if he does that? … it is our responsibility as the parents.

Notably, it was also about handling their own emotions in a stressful situation. On the one hand they had to cope with problems within the household; on the other hand, they had to cope with social pressure. Accordingly, their emotions became overwhelming.

From there I tied him, I tied [his] hands and tied [his] feet. I tied him to a coconut tree. I tied him because all the household utensils were destroyed, and my emotions arose against him at that time.

At these points in time, their own feelings came into the foreground, which prevented them from seeing the other as a person who was suffering. Even though they cared for the person, they felt powerless to cope with the behaviour other than by using pasung. Consequently, controlling the aggression became the focus. This meant that it was not the person they tied up; rather, it was the behaviours they controlled. Therefore, when not seeing the person, their actions seem to lack compassion.

Until now, he peed and defecated in the room. He never said anything especially if he was tied up and could not go anywhere, so my wife used to clean up, but I must be there, guarding that room, afraid too if something happened to my wife.

They did not see this as punishment but as the only way to manage things. Indeed, using pasung became an everyday occurrence. In other words, to avoid being involved in how the relative was feeling or suffering, the family became seemingly indifferent to the person and did not see them as someone in need of care and support.

We give him food and he eats by himself. He does not bathe because we keep him tied up unless we want to give him water [to bathe] so he can shower. If he pees and defecates, yes, he does it in that place where we tied him.

## Discussion

This article has explored families’ experiences of using pasung—illustrating its socially situated nature. In summary, the findings reveal a process that starts with not understanding a family member's behaviour, which gives rise to a growing sense of loss of the person they once knew, a dynamic that sets in train a struggle to see the other as a person with their own feelings, ideas, hopes, and needs. This is akin to a process of depersonalization of the other—that is to say, coming to see someone as lacking a personal identity. Laing (2010, p. 46) describes depersonalization as “a technique that is universally used as a means of dealing with the other when he becomes tiresome or disturbing,” which means that the person stops responding to the other's feelings as if they were dead or without subjectivity.

However, one might ask whether using pasung is just a technique. Rather, our analysis suggests that using pasung is a situated process that unfolds over time, and which increases the families’ control over what is happening. As such, using pasung is a way of trying to re-establish some stability in everyday life especially with regard to the stresses and strains of living with a family member who is mentally ill in a sociocultural context of poor support and high expectations relating to personal responsibility. Thus, according to our analysis, using pasung cannot be reduced to a simple technique. Moreover, pasung seems to emerge from a process of depersonalization when those who have authority—in this case parental authority—experience various kinds of social pressure to act and control troubling phenomena.

These social pressures intensify the emotional stress associated with everyday life with a family member whose mental health is deteriorating in some obvious ways, and the feelings of powerlessness that accompany these events. This suggests that a critical step in the process of using pasung is the manifestation of alien behaviours which obscure the everyday reality of a person struggling with mental illness. Therefore, we interpret our findings in terms of Laing's view of coercion as involving a process of depersonalization, which occurs when behaviour is challenging, but we also offer a more layered and complex interpretation. Thus, using pasung becomes a culturally accepted value system that provides guidance to families on how to react to difficult circumstances in the absence of wider support.

This then raises the question of how we can account for such moments when empathy seems to be absent (Hollan & Throop, 2008). And perhaps this is even harder because of parents’ emotional reactivity to the situation—their own involvement in the situation makes it difficult to get any distance from it and understand it in an empathic way.

In phenomenology, the most basic form of empathy acquaints you with another's experiential life where moral reflections begin in the suffering of the other person (Svenaeus, 2017). Through empathy, we connect with the other's experiential world; thus, empathy is the capacity for “feeling into” (*Einfühlung*) the other in a participatory way (Stein, 1989). However, empathy does not infer a form of fusion or “feeling of unity” as if one has a mutual experience of the same thing—empathy acknowledges that the sadness belongs to the other and is not mine (Stein, 1989).

Our findings suggest that when families perceive the other as lost and mainly as alien behaviours, they have few guidelines on how to perceive the other's lived situation. As such, there is no longer coexistence; the other is reduced to the perceived behaviours, which come to be apprehended as behaviour without meaning and subjectivity, and thus not as the person they once knew. In other words, the behaviours are alien and beyond the comprehension of the families and that is the meaning they attach to it. Depersonalization analytically captures this process.

Furthermore, the family is under social pressure and emotional stress. Thus, on the one hand, when the family is experiencing the other as alien and unknown—not recognizing the other's experiential world because of the strange behaviour—their empathy seems to reach its limits (Kirmayer, 2015). On the other hand, the prevailing sociocultural values are perceived and interpreted as making them responsible for the situation and needing to act according to that which is expected of them. In these circumstances, they do not have the capacity to feel into the other in a participatory way. Accordingly, the family's actions do not seem to be based on empathy, but rather on how they can give some stability to their world by avoiding stigma, minimizing their economic burdens, and preserving some family honour. In other words, pasung is embedded in the sociocultural values that guide the family to re-establish social order and to take control in stressful situations.

As our findings indicate, the family's stress and uneasiness are lessened when using their power (e.g., Vetlesen, 2005). This suggests that families resolve the tension between powerlessness and control by using pasung. When families are overwhelmed and impoverished by external stressors, their emotions are aroused and projected into the situation. Thus, their own emotionally charged experience obscures the subjective person with mental illness, who thus loses some of his or her humanity. This suggests that the family's actions are constrained by the sociocultural convention of doing the right thing for the community and taking responsibility for the situation. Using pasung in stressful situations might secure the safety of the family relative but at the same time worsens the conditions of their everyday life. In other words, guided and constrained by their sociocultural situation, the families struggle to make life manageable.

## Conclusion

We have used Giorgi's phenomenological method to shed light on how the use of pasung by families with a mentally ill relative emerges in a sociocultural context characterized by norms and conventions that make their everyday lives overwhelming. The phenomenological method is challenging for researchers to implement in a sustained way, because of the requirement to bracket out prior assumptions, values, and knowledge and genuinely approach the phenomenon with fresh eyes. We recognized that this may be a limitation in our study in that we may not have been sufficiently sensitive to hearing the voices of our participants and our bracketing out may have been partial. Nonetheless, we think our analysis contributes valuable insights to an important issue, which reveals the complexity of lives lived in environments where family responsibilities are significant and sources of support are limited.

We have discussed the disjunction between sociocultural demands and the family's capacity to meet these demands—the sociocultural and the psychological—in relation to using pasung. While there is always a risk of oversimplifying complicated dilemmas, our findings illustrate that the process of depersonalization is not in itself a cultural phenomenon—or just a technique. Rather, it is an analytic concept that gives meaning to the enactment of pasung that takes place in a particular sociocultural context. In summary, using pasung emerges when there is a discrepancy between demands and capacity to cope with them. Thus, the process of depersonalization that we have described is not unique to Indonesia, but rather appears in different sociocultural contexts which shape socially situated responses. Indeed, coercion is a major public mental health care concern across the globe, although different in appearance in different places. The analysis presented in this article has the potential to shed light on other forms of coercion common across differing sociocultural contexts.

This phenomenological study offers some insight into the phenomenon using pasung, which on its own cannot solve problems. Rather, it aims to understand the first-person experience of the phenomenon. There is some potential, however, for the findings to be used to inform how evidence-based mental health care services might better support families alongside protecting the human rights of relatives with psychosocial disabilities (Guan et al., 2015). In this regard, the challenge for future research is to find concrete ways of helping families act differently; that is to say, in accordance with human rights. One way forward would be to use participatory action research design, which would aim to change practice among those who are involved in the use of pasung by doing research *with* people rather than *on* them. As this study suggests, the challenge is both to change how people care for those with mental illness while at the same time changing the sociocultural context to facilitate options for choices that are grounded in human rights.
